# Improvement of Ethanol Production in *Saccharomyces cerevisiae* by High-Efficient Disruption of the *ADH2* Gene Using a Novel Recombinant TALEN Vector

**DOI:** 10.3389/fmicb.2016.01067

**Published:** 2016-07-11

**Authors:** Wei Ye, Weimin Zhang, Taomei Liu, Guohui Tan, Haohua Li, Zilei Huang

**Affiliations:** State Key Laboratory of Applied Microbiology Southern China, Guangdong Provincial Key Laboratory of Microbial Culture Collection and Application, Guangdong Open Laboratory of Applied Micrbiology, Guangdong Institute of MicrobiologyGuangzhou, China

**Keywords:** *Saccharomyces cerevisiae*, Fast TALEN technology, disruption, complement, bioethanol production

## Abstract

Bioethanol is becoming increasingly important in energy supply and economic development. However, the low yield of bioethanol and the insufficiency of high-efficient genetic manipulation approaches limit its application. In this study, a novel transcription activator-like effector nuclease (TALEN) vector containing the left and right arms of TALEN was electroporated into *Saccharomyces cerevisiae* strain As2.4 to sequence the alcohol dehydrogenase gene *ADH2* and the hygromycin-resistant gene *hyg*. Western blot analysis using anti-FLAG monoclonal antibody proved the successful expression of TALE proteins in As2.4 strains. qPCR and sequencing demonstrated the accurate knockout of the 17 bp target gene with 80% efficiency. The TALEN vector and *ADH2* PCR product were electroporated into Δ*ADH2* to complement the *ADH2* gene (*ADH2*^+^ As2.4). LC–MS and GC were employed to detect ethanol yields in the native As2.4, Δ*ADH2* As2.4, and *ADH2*^+^ As2.4 strains. Results showed that ethanol production was improved by 52.4 ± 5.3% through the disruption of *ADH2* in As2.4. The bioethanol yield of *ADH2*^+^ As2.4 was nearly the same as that of native As2.4. This study is the first to report on the disruption of a target gene in *S. cerevisiae* by employing Fast TALEN technology to improve bioethanol yield. This work provides a novel approach for the disruption of a target gene in *S. cerevisiae* with high efficiency and specificity, thereby promoting the improvement of bioethanol production in *S. cerevisiae* by metabolic engineering.

## Introduction

With the development of economics and the constant increase in energy consumption, fossil fuels could not meet the requirements of humankind. Bioethanol is becoming increasingly important in energy supply and economic development. However, the traditional bioethanol production has low conversion rate, high cost, and low yield. *Saccharomyces cerevisiae* is a key microorganism that could produce bioethanol; however, the insufficiency of high-efficient genetic manipulation methods for *S. cerevisiae* limits the wide application of bioethanol. Therefore, genetic engineering approaches with high efficiency for *S. cerevisiae* must be developed to improve bioethanol yield.

Native *S. cerevisiae* can only produce ethanol by utilizing glucose; the disadvantages of low ethanol yield and unavailability of other carbon sources, such as cellulose and starch, prevent the wide use of native *S. cerevisiae* in producing ethanol. Therefore, considerable efforts have been exerted to obtain a genetically engineered *S. cerevisiae* strain that would improve bioethanol yield. Introduction of the exogenous gene encoding xylose isomerase endows *S. cerevisiae* with the ability to utilize xylose for ethanol production (Kuyper et al., 2005; Matsushika et al., 2009). The *GDP1* gene encoding glyceraldehyde-phosphate dehydrogenase was integrated with the genome of *S. cerevisiae* to facilitate NAPDH regeneration, thereby promoting bioethanol production from xylose through the pentose pathway (Verho et al., 2003).

Aside from utilizing different carbon sources, genes involved in the ethanol production pathway were also regulated or disrupted to improve bioethanol yield. Alcohol dehydrogenase Adh2p encoded by the *ADH2* gene could catalyze ethanol into aldehyde, and the affinity of Adh2p to ethanol is approximately 10 times higher than that of other isozymes (Wiebe et al., 2007). Thus, many scholars have attempted to regulate or disrupt the expression of the *ADH2* gene. The yeast regulatory protein ADR1 could activate the expression of the *ADH2* gene; thus, the expression of *ADH2* is repressed by controlling the synthesis of the ADR1 protein (Vallari et al., 1992). Many genetic engineering approaches have been employed to delete *ADH2* in *S. cerevisiae*. However, PCR-mediated homologous recombination is only suitable for the genetic manipulation of *S. cerevisiae* with a clear genetic background (Beier et al., 1985; Gray and Honigberg, 2001). The Cre/*lox*P recombination system possesses a short homologous arm but is accompanied by a relatively low efficiency and residual *lox*P site, which may result in the knockout of non-target genes (Gueldener et al., 2002; Fang et al., 2011). Therefore, novel genetic engineering approaches with high efficiency and specificity must be developed to regulate the expression of *ADH2* in *S. cerevisiae*.

Transcription activator-like effector nuclease (TALEN) is a hybrid protein containing DNA-binding and *Fok*I cleavage domain. The DNA-binding domain consists of a variable number of tandem repeats of a 34-amino-acid monomer, which specifies the DNA-binding sequence by its 12 and 13th repeat-variable di-residues (RVDs, which specifically recognize a single nucleotide; Bogdanove and Voytas, 2011; Grau et al., 2013; Nakajima and Yaoita, 2013). TALEN technology has been widely used in the genome editing of various species, including human, plant, and zebrafish, because of its high specificity and efficiency (Joung and Sander, 2013; Sakuma et al., 2013; Zhang et al., 2013; Zu et al., 2013; Uhde-stone et al., 2014). However, only few studies have applied TALEN technology in microorganisms because of its relatively complicated reconstruction procedure for TALEN vectors. The establishment of Fast TALEN technology widens the application of TALEN in different fields (Bogdanove and Voytas, 2011; Grau et al., 2013).

In this study, a novel recombinant TALEN vector targeting the 17 bp *ADH2* gene was electroporated into *S. cerevisiae* As2.4. SDS-PAGE and Western blot were performed to confirm the successful introduction of the TALEN vector. The results of sequencing, qRT-PCR, and enzymatic activity assay demonstrated the accurate knockout of the target gene. The disruption of *ADH2* significantly improved the bioethanol yield of *S. cerevisiae* As2.4; the complement of *ADH2* was also used to confirm the function of this gene in *S. cerevisiae* As2.4. This study is the first to report on the disruption of *ADH2* in *S. cerevisiae* using Fast TALEN technology to improve bioethanol yield. The results of this study could widen the application of bioethanol and promote the development of genetic engineering in yeast.

## Materials and Methods

### Strain, Plasmid, and Growth Medium

**FIGURE 1:**
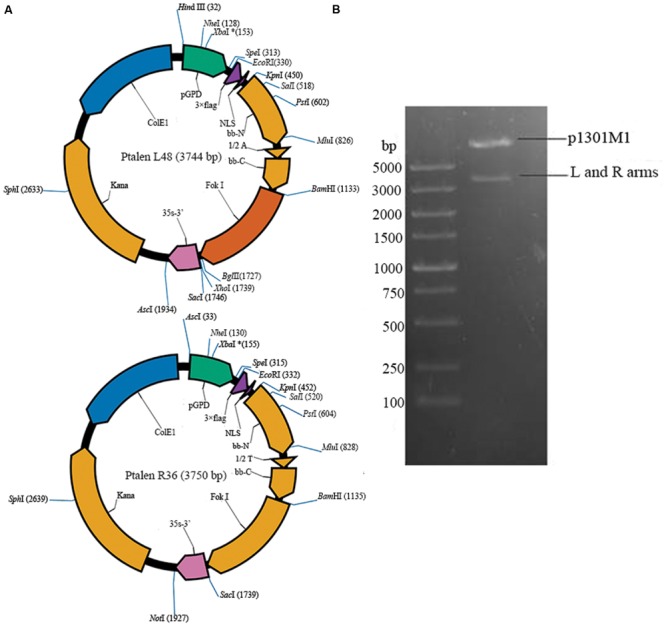
**Construction of recombinant TALEN vector. (A)** Vector map of Ptalen L48 and Ptalen R36; **(B)** Digestion of recombinant TALEN vector p1301M1-TALENs with restriction enzymes *Hin*dIII, *Sac*I, and *Asc*I.

*Saccharomyces cerevisiae* As2.4 (GIM 2.167) was provided by Microbial Culture Collection Center of Guangdong Institute of Microbiology. The Fast TALEN Assembly kit and TALEN backbone vector p1301M1 were purchased from SIDANSAI (Shanghai, China). Yeast cells were grown at 30°C in YPD medium (1% yeast extract, 2% peptone, and 2% glucose). The vector maps of Ptalen L48 and Ptalen R36 are shown in **Figure 1A**. Anti-FLAG monoclonal antibody was purchased from CST (Danvers, MA, USA). All primers used in this study are listed in **Table 1**.

**Table 1:** Primers for the construction of recombinant TALEN vectors.

Primers	Sequence
F1	5′-ggaattccatatgtctattccagaaactcaaaaag-3′
R1	5′-ggaattccatatgtctattccagaaactcaaaaag-3′
F2	5′-ctattcctttgccctcggacgag-3′
R2	5′-atgaaaaagcctgaactcac-3′
F3	5′-ccattatcttctacgaatccaacggc-3′
R3	5′-tgggctttggctttggaactgggat-3′

### Construction of Recombinant TALEN Vector

Primers F1 and R1 were used to obtain the full sequence of *ADH2*. The 17 bp target sequence of AGTTGGAGCATAAGGAT in *ADH2* was selected using E-TALEN^1^. The left and right arms of sequence-specific TALENs (Ptalen L48 and Ptalen R36) targeting 17 bp sequences adjacent to AGTTGGAGCATAAGGAT were constructed through one-step ligation using the FastTALE^TM^ TALEN Assembly Kit (SIDANSAI) in accordance with the manufacturer’s instructions. PCR and sequencing confirmed the successful construction of recombinant Ptalen L48 and Ptalen R36. Recombinant Ptalen L48 was digested with restriction enzymes *Hin*dIII and *Asc*I, and Ptalen R36 was digested with restriction enzymes *Asc*I and *Sac*I. Subcloning vector p1301 M1 containing the hygromycin phosphotransferase (*hyg*) gene was digested with *Hin*dIII and *Sac*I. The resultant larger fragments were recovered, ligated by T4 DNA ligase (Fermentas, USA) at 22°C for 16 h, and then transformed into *Escherichia coli* Top10 competent cells. Positive clones were screened via 100 μg/mL hygromycin and further confirmed through restriction enzyme digestion (*Hin*dIII, *Asc*I, and *SacI*) and sequencing.

### Introduction of Recombinant TALEN Vector

*Saccharomyces cerevisiae* As2.4 was cultivated in YPD medium and collected at the early stage of the logarithmic phase to prepare competent cells. As2.4 cells were collected at an OD_600_ of approximately 1.0 via centrifugation at 6000 rpm at 4°C. After incubation on ice for 10 min, the cells were washed twice with ice-cold sterilized water and then resuspended in sterilized water. The recombinant plasmid p1301 M1-TALENs was electroporated into As2.4-competent cells under 2.0 kV voltage and 5.0 ms pulse duration across a 2 mm cuvette. Positive clones were selected through PCR using primers F2 and R2 to amplify the *hyg* gene and further confirmed by sequencing.

The PCR products of the *ADH2* gene and recombinant plasmid p1301M1-TALENs were electroprated into Δ*ADH2* As2.4. PCR and sequencing were performed to confirm the successful complementation of *ADH2* in Δ*ADH2* As2.4 (*ADH2*^+^ As2.4).

### qPCR and Western Blot of Recombinant As2.4 Strain

After 24 h of incubation, the total RNAs of native As2.4, Δ*ADH2* As2.4, and *ADH2*^+^ As2.4 were extracted, and cDNAs were obtained using 5× All-In-One MasterMix (Abm, Canada). Primers F3 and R3 combined with cDNAs and 2× SYBR green qPCR Mix (Fermentas, USA) were used to amplify a 70 bp fragment containing the target sequence. GAPDH was employed as a reference gene. qPCR was performed in accordance with the manufacturer’s instructions. Each reaction was conducted in biological triplicate. The C_T_ values obtained were used as the original data to calculate the relative expression level of the target gene to the GAPDH gene by the 2^-ΔΔCT^ method. The qRT-PCR product was also identified by 2% agarose gel electrophoresis.

The total proteins of As2.4 strains were extracted by sonication (50 W at 3 s on, and 3 s off), and the supernatants were obtained by centrifugation at 6000 rpm at 4°C. The supernatants were loaded to 12% SDS-PAGE and then transferred to an NC membrane at a voltage of 100 V for 70 min. The membrane was blocked by 5% non-fat milk and then incubated with anti-FLAG mouse monoclonal antibody with a dilution factor of 1:2000 at 4°C overnight. The membrane was washed with TBST buffer and then incubated with rabbit anti-mouse IgG with a dilution factor of 1:2000. The target bands were visualized with an ECL kit (Thermo Fisher Scientific, USA) in accordance with the manufacturer’s instructions.

### Alcohol Dehydrogenase Activity Assay

The cells of native As2.4, Δ*ADH2* As2.4, and *ADH2*^+^ As2.4 strains were collected via centrifugation, and the proteins were released by a high-pressure cell disruption system (Constant Systems, Warwick, UK) under 1100 bar for three cycles. Supernatants were obtained after centrifugation. NAD I (5.5 mM) was dissolved in 7.5 g/L glycine buffer (pH 9.0) with a volume ratio of 1:5 as a substrate. Supernatants (5 μL) from different As2.4 strains were added to 100 μL of the substrate, and 5 μL of purified water was added as blank. After incubation at 37°C for 2 min, 20 μL of ethanol was added to the mixture and then incubated at 37°C for 5 min. The absorbance of the reaction mix at 340 nm was detected on a microplate reader (Thermo scientific, USA). Enzymatic activity was defined as 1 μg of NADH released by the catalysis of alcohol dehydrogenase in one minute (Beier et al., 1985).

### Analysis of Ethanol Yield in As2.4 Strains

The native As2.4, Δ*ADH2* As2.4, and *ADH2*^+^ As2.4 strains were inoculated into 150 mL of YPD medium in a 250 mL flask and then cultivated at 30°C at 180 rpm. After 72 h of cultivation, the fermentation liquid was loaded onto LC–MS (Agilent 6430, American) at a flow rate of 0.2 mL/min using C18 column (2 mm × 150 mm).

Fermentation liquids from the different As2.4 strains in head-space bottles were incubated at 80°C for 30 min. After reaching the equilibrium, a predetermined amount of the head-space of the vial was flushed into the gas chromatograph; isopropanol mixed with ethanol in different ratios was employed as the internal standard. Ethanol yield was identified according to the peak areas of test samples and standard substances. Each analysis was carried out in triplicates.

## Results

### Construction of p1301 M1-TALENs

The nucleotide sequences encoding repeats of 34 amino acids were assembled using Fast TALEN Assembly kit, inserted into Ptalen L48 and Ptalen R36 (**Figure 1A**), and then further confirmed by sequencing and restriction enzyme digestion. Digested TALEN arms were ligated with the backbone vector p1301M1. Two bands of approximately 8600 and 4000 bp were obtained after the recombinant plasmid was digested with *Hin*dIII, *Sac*I, and *Asc*I, which demonstrated the successful construction of p1301M1-TALENs (**Figure 1B**).

### Introduction of p1301M1-TALENs

**FIGURE 2:**
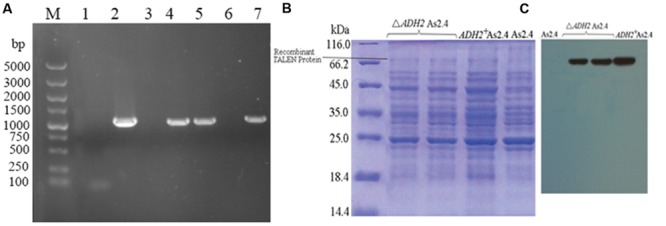
**Introduction of recombinant TALEN vector. (A)** Amplification of the *hyg* gene in Δ*ADH2* As2.4 and *ADH2*^+^ As2.4: Lane 1, PCR product without a template; Lane 2, PCR product using *hyg* gene fragment as a template; Lanes 3–7, PCR products using genomes of recombinant As2.4 colonies picked from YPD medium as templates. **(B)** SDS-PAGE analysis of total proteins from different As2.4 strains. **(C)** Western blot analysis of total proteins from different As2.4 strains using anti-FLAG antibody.

Hygromycin was used to select positive clones, and the successful amplification of approximately 1000 bp *hyg* gene indicated the successful introduction of p1301 M1-TALENs into the native *S. cerevisiae* strain As2.4 (**Figure 2A**). The acquirement of *hyg* and *ADH2* demonstrated the successful introduction of p1301M1-TALENS combined with *ADH2* into Δ*ADH2* As2.4.

The proteins of native As2.4, Δ*ADH2* As2.4, and *ADH2*^+^ As2.4 were extracted, and the supernatants were loaded onto 12% SDS-PAGE. As shown in **Figure 2B**, bands of approximately 68 kD corresponding to repeats consisting of 34 amino acids were detected in the Δ*ADH2* As2.4 strain and complement strain, whereas no corresponding band was detected in native As2.4.

As shown in **Figure 2C**, when anti-FLAG monoclonal antibody was used to perform Western blot analysis, target bands of 68 kD were also visualized in Δ*ADH2* As2.4 and *ADH2*^+^ As2.4, whereas no corresponding band was detected in native As2.4. The result further confirmed the successful introduction of p1301M1-TALENs into the As2.4 strains.

### Disruption of *ADH2* in As2.4

**FIGURE 3:**
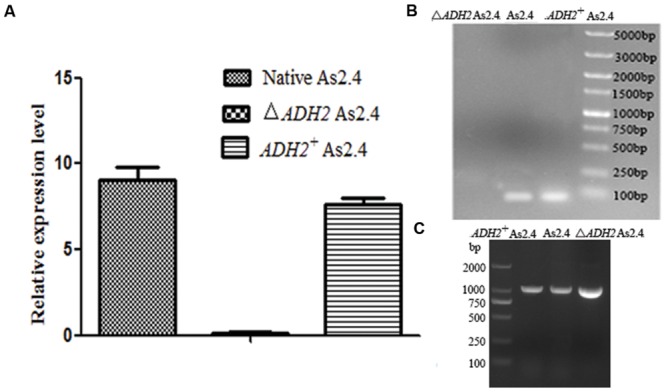
**Disruption and complementation of the *ADH2* gene in As2.4 strains. (A)** Relative expression level of 70 bp target product in different As2.4 strains. **(B)** Detection of qRT-PCR products by agarose gel electrophoresis. **(C)** Amplification of the *ADH2* gene using genomes of native As2.4, Δ*ADH2* As2.4, and *ADH2*^+^ As2.4 strains as templates.

**FIGURE 4:**
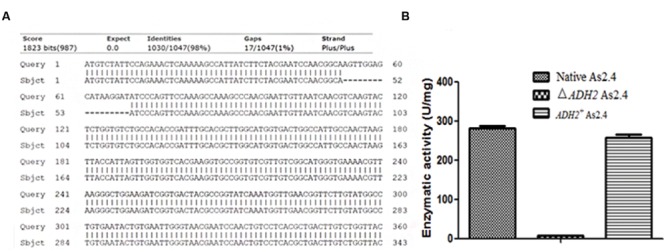
**Knockout of 17 bp target sequence. (A)** Sequence alignments of *ADH2* gene and PCR products of Δ*ADH2* As2.4. **(B)** Alcohol dehydrogenase activities of supernatants from different As2.4 strains after cell disruption.

The total RNAs of native As2.4, Δ*ADH2* As2.4, and *ADH2*^+^ As2.4 were extracted to amplify cDNAs. Primers F3 and R3 were used to amplify a 70 bp fragment containing the target sequence. As shown in **Figure 3A**, results of qRT-PCR analysis indicated that the relative expression levels of the target sequences were much higher in native As2.4 and *ADH2*^+^ As2.4 than in Δ*ADH2* As2.4. The 70 bp bands were detected in the qRT-PCR products of native As2.4 and *ADH2*^+^ As2.4, whereas, no corresponding band was detected in Δ*ADH2* As2.4 (**Figure 3B**). When F1 and R1 were used to perform PCR, a slightly smaller fragment was detected in Δ*ADH2* As2.4 compared with those in native As2.4 and *ADH2*^+^ As2.4 (**Figure 3C**). The relative expression levels in native As2.4 and *ADH2*^+^ As2.4 were also much higher than that in Δ*ADH2* As2.4. The PCR products using primers F1, R1, and genomes of native As2.4, Δ*ADH2* As2.4, and *ADH2*^+^ As2.4 were sequenced, and alignment results indicated that the 17 bp target sequence AGTTGGAGCATAAGGAT was knocked out accurately (**Figure 4A**). Twenty colonies on YPD medium were selected, and the *ADH2* gene fragment was amplified and sequenced; the target sequence in 16 colonies was knocked out successfully, indicating that *ADH2* in As2.4 was knocked out using TALEN technology with 80% efficiency.

The alcohol dehydrogenase activities of supernatants from the different As2.4 strains were assayed, as shown in **Figure 4B**; supernatants of native As2.4, Δ*ADH2* As2.4, and *ADH2*^+^ As2.4 showed enzymatic activities of 282 ± 10.3, 5.6 ± 0.5, and 258 ± 12.5 U/mg, respectively. The results indicated that Δ*ADH2* As2.4 rarely showed any alcohol dehydrogenase activity after *ADH2* in As2.4 was disrupted by the recombinant TALEN vector.

### Ethanol Yields in Different As2.4 Strains

**FIGURE 5:**
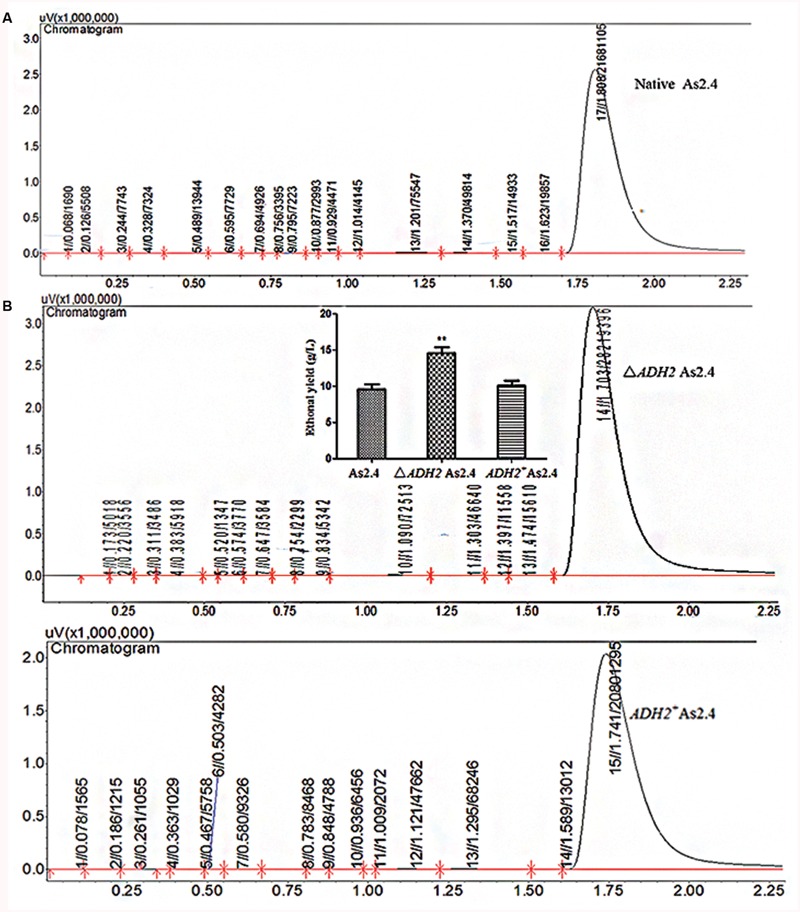
**Detection of ethanol yields in different As2.4 strains. (A)** Detection of ethanol in As2.4 strains by gas chromatography. **(B)** Ethanol yields in native As2.4, Δ*ADH2* As2.4, and *ADH2*^+^ As2.4.

The ethanol yields in the different As2.4 strains were detected by LC–MS and GC. As shown in **Supplementary Figure S1**, the anion peak of 45.0 corresponding to ethanol was detected in fermentation liquids in the different As2.4 strains and ethanol standards. This result indicates that ethanol was successfully produced by the different As2.4 strains. The peak area of ethanol in Δ*ADH2* As2.4 was significantly larger than those in native As2.4 and *ADH2*^+^ As2.4 (**Figure 5A**). The ethanol yields in native As2.4, Δ*ADH2* As2.4, and *ADH2*^+^ As2.4 were 9.6 ± 1.51, 14.6 ± 1.35, and 10.1 ± 1.22 g/L, respectively, according to the peak areas of different internal standard substances. Ethanol production was improved by 52.4 ± 5.3% through the disruption of *ADH2* in As2.4 and decreased to the same level as that in native As2.4 after *ADH2* was complemented into Δ*ADH2* As2.4 (**Figure 5B**).

## Discussion

In this study, the *ADH2* gene in As2.4 was first disrupted using Fast TALEN technology. The ethanol yield in As2.4 was significantly improved by the disruption of *ADH2*. The illustration of improvement of ethanol yield in *S. cerevisiae* strain using Fast TALEN technology was shown in **Supplementary Figure S2**. The results of this study would lay a foundation for the metabolic engineering of *S. cerevisiae*, thereby promoting the application of bioethanol in different industries.

Alcohol dehydrogenase encoded by *ADH2* could catalyze ethanol to aldehyde. In consequence, different approaches, including PCR-mediated homologous recombination and Cre-*lox*P system, were employed to knock out *ADH2* and improve ethanol yield (Ding et al., 2014; Zheng et al., 2015). However, these approaches showed low efficiency, low specificity, and detectable alcohol dehydrogenase II activity in *ADH2* gene depletion strain (Wang et al., 2009). Moreover, ethanol yield was only improved by 5–20% through the disruption of *ADH2*. The relatively lower improvement probably resulted from the residual alcohol dehydrogenase II activity after *ADH2* was knocked out (Wang et al., 2009; Krivoruchko et al., 2011). In the present study, *ADH2* in As2.4 was disrupted by a novel recombinant TALEN vector with 80% efficiency, and the enzymatic activity assay results revealed that the total proteins from Δ*ADH2* As2.4 rarely showed any alcohol dehydrogenase activity toward alcohol. Moreover, the sequencing result revealed no off-target effect during the knockout of *ADH2* in native *S. cerevisiae* strain As2.4. The ethanol yield in As2.4 was improved by 52.4 ± 5.3% through the disruption of *ADH2*; the increasing ratio of ethanol yield was much higher than those previously reported (Wang et al., 2009; Krivoruchko et al., 2011). *ADH2* was also knocked into Δ*ADH2* As2.4 using Fast TALEN technology. The bioethanol level produced in *ADH2*^+^ As2.4 was almost the same as that in native As2.4, which further demonstrated the function of *ADH2* during bioethanol production in *S. cerevisiae* strain. This Fast TALEN technology for the disruption of *ADH2* in native As2.4 showed high efficiency and specificity compared with previously reported methods.

## Conclusion

In this study, *ADH2* in *S. cerevisiae* As2.4 strain was disrupted and complemented. Ethanol yield was improved by 52.4 ± 5.3% through the disruption of *ADH2* in As2.4 using Fast TALEN technology. The complement of *ADH2* could decrease the ethanol yield in *ADH2*^+^ As2.4 to the same level as that in native As2.4. To the best of our knowledge, this study is the first to report on the disruption of a target gene in *S. cerevisiae* using Fast TALEN technology. This study would provide a novel approach for the disruption of a target gene in *S. cerevisiae* with high efficiency and specificity, thereby promoting the improvement of ethanol production in *S. cerevisiae* by metabolic engineering.

## Author Contributions

WY: design the experiment, WZ: direct the experiment, WY, TL, GT, HL, ZH: did the experiment.

## Conflict of Interest Statement

The authors declare that the research was conducted in the absence of any commercial or financial relationships that could be construed as a potential conflict of interest.

## Footnotes

1 http://www.e-talen.org

## Supplementary Material

FIGURE S1 **Detection of ethanol yields in native As2.4, Δ*ADH2* As2.4, and *ADH2*^+^ As2.4 strains by LC–MS**.

FIGURE S2 **The illustration of improvement of ethanol yield in *S. cerevisiae* strain using Fast TALEN technology**.
