# Supplementary figures and images for: Improvement of Ethanol Production in Saccharomyces cerevisiae by High-Efficient Disruption of the ADH2 Gene Using a Novel Recombinant TALEN Vector
Source: Front Microbiol. 2016 Jul 11;7:1067. doi: 10.3389/fmicb.2016.01067 (PMC4939295; doi:10.3389/fmicb.2016.01067)

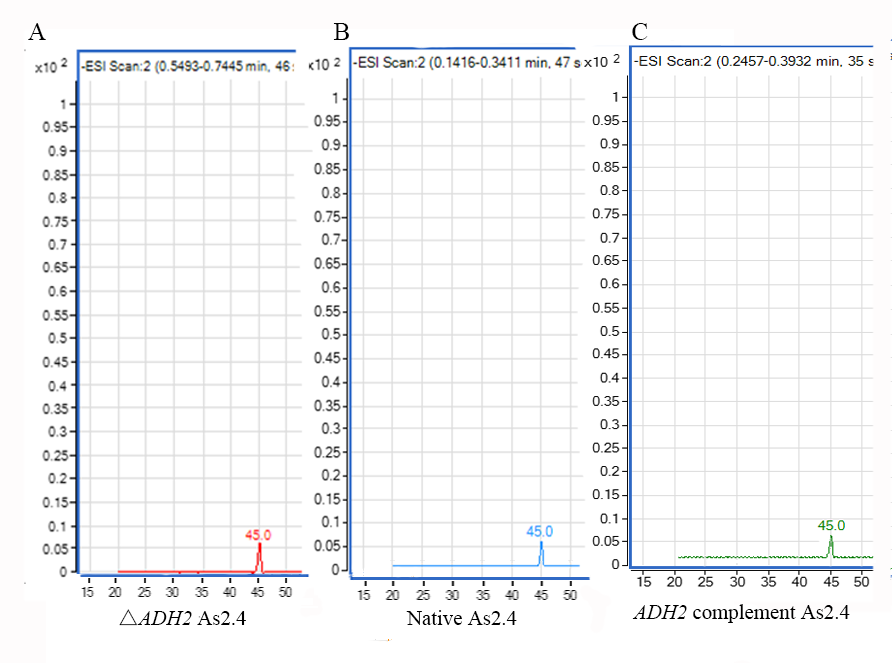

Supplement: FIGURE S1 — Detection of ethanol yields in native As2.4, ΔADH2 As2.4, and ADH2+ As2.4 strains by LC–MS. [file Image_1.TIF]

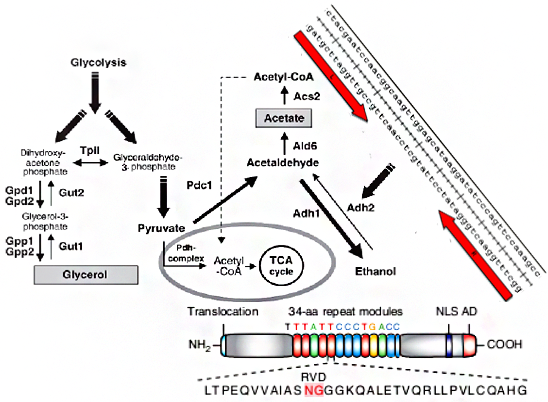

Supplement: FIGURE S2 — The illustration of improvement of ethanol yield in S. cerevisiae strain using Fast TALEN technology. [file Image_2.TIF]
